# Trust in Physicians Exceeds Trust in Social Media, Family, and Friends for Health Information Among Adult Social Media Users

**DOI:** 10.7759/cureus.117935

**Published:** 2026-10-14

**Authors:** Matthew Jervis, France Nguyen-Grozavu, Aaron Jacobs

**Affiliations:** 1 Medicine, California University of Science and Medicine, Colton, USA; 2 Epidemiology and Public Health, Herbert Wertheim School of Public Health and Human Longevity, University of California San Diego, La Jolla, USA; 3 Medical Education, California University of Science and Medicine, Colton, USA

**Keywords:** digital health media, health information, health literacy, physician trust, public health, social media

## Abstract

Social media has dramatically expanded access to health information beyond traditional clinical settings. Patients increasingly turn to digital platforms for immediate health-related information. While this democratization of information has possible benefits, such as increased health literacy, it also raises concerns about misinformation and erosion of trust in healthcare professionals trained to address these questions.

The primary objective of this study is to quantify levels of trust in differing sources of health information among adult social media users in the US. We additionally evaluate whether time spent on social media is associated with trust in physician-delivered health information. We hypothesize that physicians remain the most trusted source of health information among the three sources evaluated. Additionally, we hypothesize that greater time spent on social media is associated with lower trust in physicians.

A cross-sectional survey study was conducted among adults 18 years or older who reported social media use within the past seven days and had visited a physician in the US within the past one year. Social media use was limited to the following platforms: Facebook, YouTube, TikTok, Twitter/X, Reddit, LinkedIn, and Instagram.

Among the 65 participants, most were female, White, held a bachelor’s degree, and were between 25 and 30 years of age. Trust in health information differed significantly across information sources. Participants reported the greatest trust in physicians, followed by family or friends, and the lowest trust in social media (*p* < 0.05). No significant differences were observed between users and non-users of each social media platform with regards to trust in physician-delivered health information. Additionally, time spent on social media was not associated with trust in physician-delivered health information.

Despite widespread social media use among participants, physicians remained the most trusted source of health information. This underscores physicians' enduring role as a trusted source in the digital age. Future research can investigate the factors that sustain this trust and further influences on health-related decision-making and behaviors.

## Introduction

The traditional model of patients seeking healthcare advice directly from physicians is undergoing a rapid transformation. In the past, physicians have served as the primary and often most trusted source for medical information [1]. However, with the proliferation of readily accessible online platforms such as Instagram, TikTok, and other social media channels over the past 25 years, patients now increasingly turn to these sources for immediate health-related information [2-4]. A 2024 health consumer survey of 4,012 US adults found that 52% who tried a health approach, trend, or product in the last year learned about it through a social media platform [5]. Despite concerns about the reliability of COVID-19-related information on social media, 63.6% of social media users were unlikely to verify what they read online with a health professional during the pandemic [6]. While democratization of information access may contribute to increased health literacy, it also raises concerns about potential misinformation, the deconstruction of medical advice, and erosion of trust in healthcare professionals [7-9]. Medical practice relies not solely on delivering evidence-based information but also on individualized clinical judgment. The delivery of nuanced patient care can be undermined by fragmented or generalized health information found online, especially when published by physicians themselves [10].

Current literature is mixed with regards to the effect of social media and trust in physicians for health information, but patient trust in physicians overall remains high [4,11]. A recent study using Health Information National Trends Survey (HINTS) data showed that those who perceive substantial health misinformation on social media have higher odds of reporting low trust in the healthcare system [12]. Using a cross-sectional survey design, this study evaluates associations between social media use, trust in physician-delivered health information, and trust in other common sources of health information.

This study aims to quantify levels of trust adults place in health information delivered by differing sources, including physicians, family and friends, and social media. Additionally, this study seeks to examine if there is a correlation between time spent on social media and trust in health information delivered by physicians. We hypothesize that physicians remain the most trusted source of health information among the three sources evaluated and that greater time spent on social media is associated with lower trust in physician-delivered health information.

## Materials and methods

A cross-sectional survey study was conducted between winter 2025 and spring 2026 to evaluate health information-seeking behaviors and trust in health information sources among adult social media users in the US. Participants were recruited using convenience sampling through social media outreach on Instagram and Facebook, classroom-based recruitment at local universities, and flyer advertisements in Orange County, California. Participation was voluntary and anonymous. The California University of Science and Medicine Institutional Review Board determined this study to be exempt.

Eligible participants were adults aged 18 years or older who reported using at least one listed social media platform within the previous seven days and who had visited a physician in the US within the previous year. Individuals who did not meet these criteria were excluded from analysis. A total of 72 responses were received, of which seven were excluded for failure to meet inclusion criteria, resulting in a final analytic sample of 65 participants. Participant recruitment, exclusion, and study flow are summarized in Figure 1.

**Figure 1:**
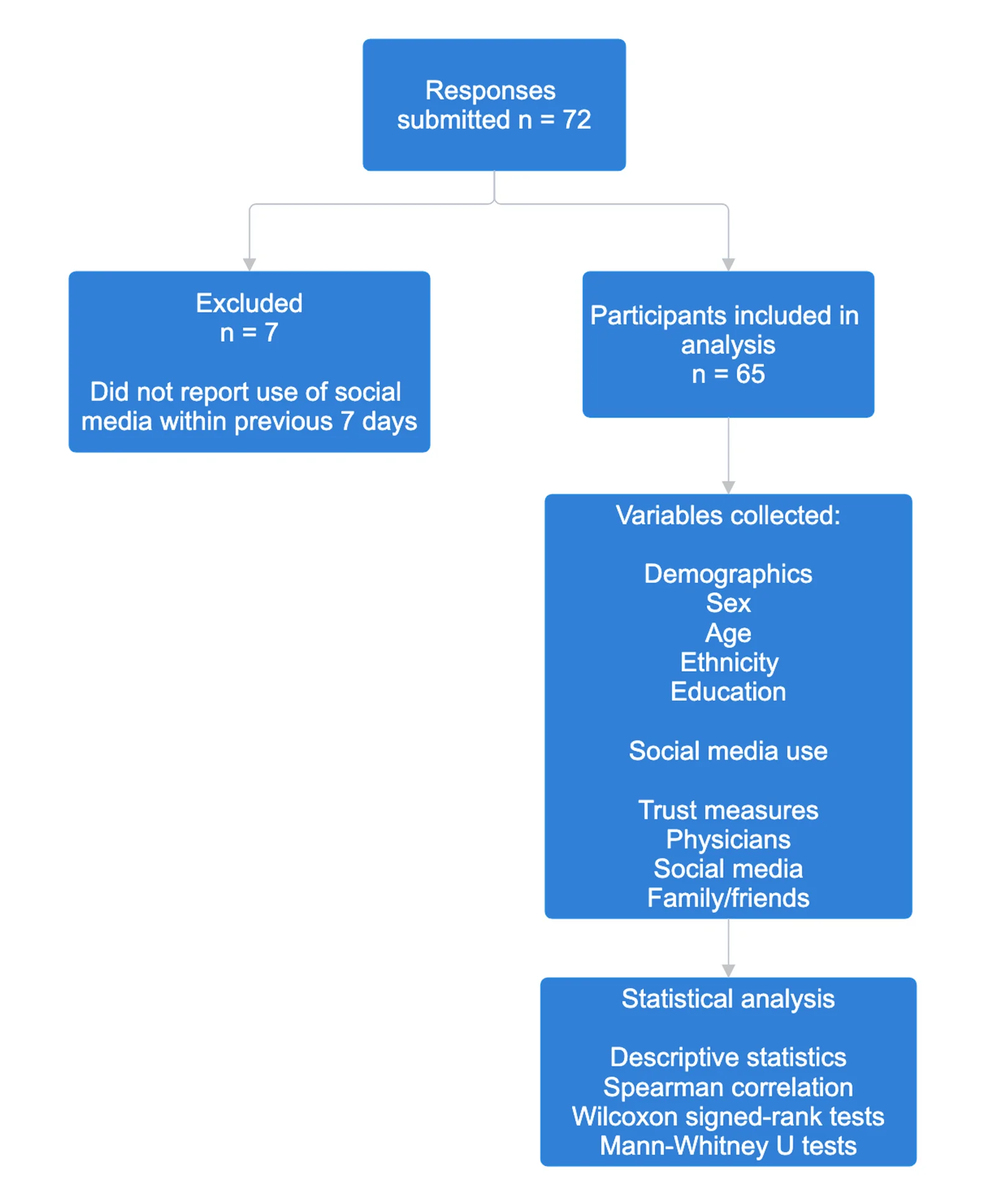
Data collection flowchart

Survey instrument

Participants completed an electronic survey delivered via Google Forms consisting of demographic, social media use, and health information trust questions on a Likert scale. Participants were asked to identify which social media platforms they had used during the previous seven days from a predefined list of common platforms (Facebook, YouTube, TikTok, Twitter/X, Reddit, LinkedIn, and Instagram). For each platform used, participants estimated their average daily time spent on the platform using ordinal response categories ranging from 0 hours to at least 8 hours per day.

Trust in health information delivered by physicians, family or friends, and social media was assessed using identical four-point Likert scale items. Survey items were developed by the study team and informed by the HINTS, prior literature, and the study objectives. Participants were asked how much they trusted health or medical information obtained from each source and selected one of the following responses: "A lot" (4), "Some" (3), "A little" (2), or "Not at all" (1). The full survey instrument is provided in Appendix A.

Statistical analysis

IBM SPSS Statistics Version 28.0.1.0 (IBM Corp., USA) was used for data analysis. Descriptive statistics were calculated for participant demographic characteristics, healthcare utilization, social media platform use, and trust measures. Pairwise Wilcoxon signed-rank tests compared trust in health-information delivered by a physician, family and friends, and social media. Mann-Whitney U tests compared trust in physician-delivered health-information between users and non-users of each platform. Spearman correlations examined time spent on individual social media platforms compared to trust in physician-delivered health-information among participants who reported using the respective platform during the preceding seven days. Self-reported daily hours spent across the seven platforms were summed for each participant to create an aggregate exposure measure, with responses of “8+ hours” coded as 8. Spearman correlation examined the association between total self-reported daily platform hours and trust in physician-delivered health information. Two participants whose sums exceeded 24 hours were excluded from the aggregate correlation analysis. A sensitivity analysis was performed which retained all participants. All survey questions required a response before participants could proceed and no item-level data were missing from submitted surveys.

## Results

Demographics

A total of 65 participants met inclusion criteria and completed the survey per Table 1. Participants were predominantly female (n = 36, 55.4%), White (n = 24, 36.9%), and nearly half aged 25-30 years (n = 31, 47.7%). Most respondents held at least a bachelor’s degree (n = 54, 83%) and reported seeing a primary care provider (PCP) within the previous year (n = 59, 90.8%). Less than half visited a specialist physician or both a PCP and specialist physician during the same period.

**Table 1:** Participant demographic and healthcare characteristics (N = 65) PCP: Primary care provider

Characteristic	n (%)
Sex at Birth	
Female	36 (55.4)
Male	29 (44.6)
Age Group (years)	
18–24	19 (29.2)
25–30	31 (47.7)
31–40	2 (3.1)
41–50	3 (4.6)
51–60	7 (10.8)
≥61	3 (4.6)
Ethnicity	
White	24 (36.9)
Asian	21 (32.3)
Hispanic, Latin, or Spanish origin	8 (12.3)
American Indian	1 (1.5)
Middle Eastern or North African	4 (6.2)
Black or African heritage	3 (4.6)
Native Hawaiian or Other Pacific Islander	3 (4.6)
Other	1 (1.5)
Education	
High school diploma	3 (4.6)
Some college	7 (10.8)
Associate degree	1 (1.5)
Bachelor's degree	39 (60.0)
Master's degree	14 (21.5)
Doctoral degree	1 (1.5)
Healthcare Utilization (Past Year)	
Seen a PCP	59 (90.8)
Seen a specialist physician	29 (44.6)
Seen both a PCP and specialist	23 (35.4)

Participants responded which platforms they utilized in the previous seven days. YouTube (n = 53, 81.5%) and Instagram (n = 51, 78.5%) were the most frequently reported platforms, followed by Facebook (n = 40, 61.5%), and TikTok (n = 37, 56.9%) as shown in Table 2.

**Table 2:** Social media use by platform among participants in the previous 7 days

Platform	n (%)
YouTube	53 (81.5)
Instagram	51 (78.5)
Facebook	40 (61.5)
TikTok	37 (56.9)
Twitter/X	32 (49.2)
LinkedIn	32 (49.2)
Reddit	30 (46.2)

On the four-point trust scale (1 = not at all; 4 = a lot), mean (SD) trust ratings were 3.5 (0.64) for physicians, 2.6 (0.61) for family or friends, and 2.4 (0.91) for social media. Wilcoxon signed-rank testing demonstrated significant differences in trust across information sources per Table 3. Participants reported significantly greater trust in physicians than in social media sources (Z = -5.960, p < 0.001, r = 0.74) and significantly greater trust in physicians than in family or friends (Z = -5.833, p < 0.001, r = 0.72). Trust in family or friends was also significantly greater than trust in social media (Z = -2.036, p = 0.042, r = 0.25).

**Table 3:** Pairwise comparison of trust in health information sources: Wilcoxon signed-rank tests (N= 65) Effect size r was calculated as |Z|/√Nn .

Comparison	Standardized Test Statistic (Z)	p-value	Effect Size (r)
Trust in Physicians vs Trust in Social Media	-5.96	<0.001	0.74
Trust in Physicians vs Trust in Family/Friends	-5.833	<0.001	0.72
Trust in Family/Friends vs Trust in Social Media	-2.036	0.042	0.25

Mann-Whitney U testing demonstrated no statistically significant differences in trust in physician-delivered health information between users and non-users of Facebook, YouTube, Twitter/X, LinkedIn, Instagram, Reddit, or TikTok (all p > 0.05) (Table 4).

**Table 4:** Association between specific social media platform use and trust in physician-delivered health information

Platform	Mann-Whitney U	Standardized Test Statistic (Z)	p-value
Facebook	501	0.016	0.988
YouTube	308	-0.195	0.845
Twitter/X	532	0.061	0.952
LinkedIn	481	-0.712	0.476
Instagram	441	1.548	0.122
Reddit	559	0.517	0.605
TikTok	523	0.076	0.939

Spearman rank-order correlation analyses demonstrated no statistically significant associations between time spent on individual social media platforms and trust in physician delivered health information across all platforms shown in Table 5. Correlation coefficients ranged from -0.234 for Reddit to 0.186 for Instagram. An additional analysis evaluated the association between combined self-reported daily social media hours and trust in physician-delivered health information. After excluding two participants whose sums exceeded 24 hours, no statistically significant association was detected (Spearman’s ρ = −0.048, p = 0.706; n = 63). A sensitivity analysis including all 65 participants yielded a similar result (ρ = −0.053, p = 0.675).

**Table 5:** Spearman correlations between time spent on social media platforms and trust in physician-delivered health information

Platform	Spearman’s ρ	p-value	n
Facebook	-0.084	0.606	40
YouTube	-0.010	0.942	53
Twitter/X	0.095	0.606	32
LinkedIn	-0.094	0.609	32
Instagram	0.186	0.192	51
Reddit	-0.234	0.213	30
TikTok	0.149	0.379	37

## Discussion

Physicians remained the most trusted source of health information despite widespread use of various social media platforms (Table 3). Participants reported significantly greater trust in physicians than in either social media or family and friends as sources of health information with social media trusted the least. Contrary to our initial hypothesis, time spent on social media was not associated with differences in trust in physician-delivered health information, nor did trust differ between users and non-users of individual social media platforms (Tables 4, 5). Previous literature supports the nuance that it is the perception of substantial misinformation, not social media use itself, that is associated with lower trust in the healthcare system [12]. While social media has become a common source for health information, trust in physician-delivered health information was not significantly associated with use of any specific social media platform or time spent on these platforms.

Recent analyses utilizing the HINTS have similarly demonstrated that physicians remain the most trusted source of health information among US adults [11], including among underserved and vulnerable populations [13]. Another study reported the same pattern of trust observed in our study, with the highest for physicians, family and friends, and social media respectively [14]. Despite this, most Americans who utilized social media for health information were unlikely to fact-check social media content with a health professional [6]. Prior work contextualizes that convenience and access barriers to traditional healthcare are key drivers of turning to social media for health information, and that young adults are especially motivated to use these platforms due to their accessibility and relatability [15,16].

Together with our findings, this supports the conclusion that despite social media being a highly utilized source for health information, physicians remain the most trusted for health information. These findings underscore the continued importance of the physician-patient relationship, as well as reinforce the opportunity physicians have to partner with patients to contextualize health information obtained from social media.

Limitations of this study include a small convenience sample, cross-sectional design, self-reported data, and possible recall bias. The sample predominantly included younger, educated participants, limiting generalizability. Recruitment through social media, university classrooms, and local flyers may have introduced selection bias. The questionnaire was not pretested or formally evaluated for validity and reliability, which may limit confidence in the measurement of trust. The final sample size was smaller than the target determined by the a priori power calculation, potentially limiting the ability to detect associations. Summed self-reported platform hours used in the Spearman correlation analysis may overestimate distinct daily social media time as participants may use platforms simultaneously. Conversely, coding “8+ hours” as 8 may underestimate use at the upper end. The shift from questions about platform use during the previous seven days to estimates of average daily hours may have caused confusion about the reporting time frame and contributed to sums exceeding 24 hours in two participants, although this explanation could not be confirmed. Future work should include larger, more representative populations and evaluate newer digital information sources such as artificial intelligence.

## Conclusions

Despite the use of various sources for health information, physicians remain the most trusted source for health information. Social media use and time spent on social media were not significantly associated with reduced trust in physician-delivered health information. Physicians should continue strengthening this trust through patient education and evidence-based counseling. Additional research is needed in larger, more diverse populations to examine emerging digital health technologies.

## Appendices

Appendix A

Survey Instrument

Eligibility Questions:

Have you used one of the following social media platforms in the past seven (7) days?

Facebook

YouTube

Twitter/X

LinkedIn

Instagram

Reddit

TikTok

Yes - proceed

No - survey stops

Have you been seen by a physician in the United States in the past one (1) year?

Yes - proceed

No - survey stops

Survey Questions:

Sex at Birth

- Male

- Female

Age (in years)

- 18-24

- 25-30

- 31-40

- 41-50

- 51-60

- 61-70

- 71 or greater

Ethnicity (check all that apply)

- White

- Asian

- American Indian

- Black or African Heritage

- Middle Eastern or North African

- Hispanic, Latin, or Spanish origin

- Native Hawaiian or Other Pacific Islander

- Alaska Native

- Other

Country of Residence

- United States of America

- Other

Highest Level of Education Attained

- Less than high school diploma

- High school

- Some college

- Associate's degree

- Bachelor's degree

- Master's degree

- Doctoral degree

Which type of physician have you seen in the past one (1) year? Check all that apply.

Primary care physician (family medicine doctor, internal medicine doctor, general

practitioner)

Specialty physician (allergist/immunologist, rheumatologist, gastroenterologist,

psychiatrist, dermatologist, surgeon etc.)

Which of the following have you used for medical information in the past one (1) year? Check all that apply.

Social media

Physicians

Other internet sources

Friends or family

Which social media platform have you used in the past seven (7) days? Check all that apply:

- Facebook

- YouTube

- Twitter/X

- LinkedIn

- Instagram

- Reddit

- TikTok

- None of these

Estimate the average amount of time (in hours) you spend on “insert platform here”

daily.

- 0 hours to 8+ hours (scale)

In general, how frequently do you use social media when seeking medical information?

A lot

Some

A little

Not at all

In general, how much would you trust information about health or medical topics from a

doctor?

A lot

Some

A little

Not at all

In general, how much would you trust information about health or medical topics from

family or friends?

A lot

Some

A little

Not at all

In general, how much would you trust information about health or medical topics from

social media?

A lot

Some

A little

Not at all

Your responses have been recorded. Thank you for your participation.
